# Characterization of Palytoxin Binding to HaCaT Cells Using a Monoclonal Anti-Palytoxin Antibody

**DOI:** 10.3390/md11030584

**Published:** 2013-02-26

**Authors:** Marco Pelin, Sabrina Boscolo, Mark Poli, Silvio Sosa, Aurelia Tubaro, Chiara Florio

**Affiliations:** 1 Department of Life Sciences, University of Trieste, Via A. Valerio 6, 34127 Trieste, Italy; E-Mails: marco.pelin@hotmail.it (M.P.); sabribos@hotmail.com (S.B.); silvio.sosa@econ.units.it (S.S.); florioc@units.it (C.F.); 2 U.S. Army Medical Research Institute of Infectious Diseases, Ft Detrick, MD 21701, USA; E-Mail: mark.poli@us.army.mil

**Keywords:** palytoxin, ouabain, binding, ELISA, HaCaT cells

## Abstract

Palytoxin (PLTX) is the reference compound for a group of potent marine biotoxins, for which the molecular target is Na^+^/K^+^-ATPase. Indeed, ouabain (OUA), a potent blocker of the pump, is used to inhibit some PLTX effects *in vitro*. However, in an effort to explain incomplete inhibition of PLTX cytotoxicity, some studies suggest the possibility of two different binding sites on Na^+^/K^+^-ATPase. Hence, this study was performed to characterize PLTX binding to intact HaCaT keratinocytes and to investigate the ability of OUA to compete for this binding. PLTX binding to HaCaT cells was demonstrated by immunocytochemical analysis after 10 min exposure. An anti-PLTX monoclonal antibody-based ELISA showed that the binding was saturable and reversible, with a *K_d_* of 3 × 10^−10^ M. However, kinetic experiments revealed that PLTX binding dissociation was incomplete, suggesting an additional, OUA-insensitive, PLTX binding site. Competitive experiments suggested that OUA acts as a negative allosteric modulator against high PLTX concentrations (0.3–1.0 × 10^−7^ M) and possibly as a non-competitive antagonist against low PLTX concentrations (0.1–3.0 × 10^−9^ M). Antagonism was supported by PLTX cytotoxicity inhibition at OUA concentrations that displaced PLTX binding (1 × 10^−5^ M). However, this inhibition was incomplete, supporting the existence of both OUA-sensitive and -insensitive PLTX binding sites.

## 1. Introduction

Palytoxin (PLTX) is the reference compound for a group of potent marine biotoxins that have recently become endemic to the Mediterranean Sea. It was characterized for the first time in 1971 from zoanthid corals belonging to the *Palythoa* genus [1]. Later, PLTX and analogues were isolated from dinoflagellates of the *Ostreopsis* genus [2,3,4,5,6,7], as well as from other zoanthid corals [8,9,10]. Recently, a possible cyanobacterial origin of the toxin has been hypothesized [11].

Human poisonings attributed to PLTX are usually associated with ingestion of contaminated seafood and to seawater aerosol exposure during *Ostreopsis* blooms. However, dermatological problems have recently been associated with cutaneous exposure to seawater during *Ostreopsis* blooms, as well as after handling of *Palythoa* corals in home aquaria [12].

Currently, it is widely accepted that the molecular mechanism of action of PLTX resides in its interaction with the α-β heterodimer of the Na^+^/K^+^-ATPase. This interaction causes a consistent intracellular ionic imbalance due to the transformation of the pump into a non-specific cationic channel [13,14]. In accordance with this proposed mechanism of action, several studies reported the ability of ouabain (OUA), a known blocker of Na^+^/K^+^-ATPase, to inhibit PLTX effects *in vitro* [15,16,17,18,19].

The ability of PLTX to interact with Na^+^/K^+^-ATPase was demonstrated in the early 80’s through the ability of ouabain to prevent the toxin-induced lysis of erythrocytes, an effect consistently reported in several studies [20,21,22,23,24,25]. The high sensitivity of erythrocytes to PLTX made these cells a good tool to investigate the binding properties of PLTX. Indeed, ^125^I radiolabeled PLTX was reported to bind in a rapid and reversible manner to intact human erythrocytes with a *K_d_* of 2 × 10^−11^ M [26]. The binding was enhanced by divalent cations and borate and inhibited by K^+^ and OUA, the latter showing an apparent competitive behavior against PLTX on the Na^+^/K^+^-ATPase [26]. However, the incomplete reversal of PLTX biological activities by ouabain suggests a more complex interaction between PLTX and OUA at the Na^+^/K^+^-ATPase binding site. Indeed, despite the use of OUA as inhibitor/displacer of PLTX on erythrocytes and/or on purified Na^+^/K^+^-ATPase [27], it has been suggested that the OUA binding site on Na^+^/K^+^-ATPase could be distinct from that of PLTX [21,22]. Furthermore, Artigas and Gadsby [13] demonstrated that PLTX and OUA could simultaneously bind to Na^+^/K^+^-ATPase, suggesting two different co-existing binding sites on the pump.

The present study was carried out to investigate the binding properties of PLTX on living cells and to characterize the behavior of OUA as a PLTX displacer and antagonist of PLTX-induced cytotoxicity. To this end, we carried out binding experiments using an anti-PLTX monoclonal antibody-based immunoenzymatic assay (ELISA) and a cytotoxicity assay on intact HaCaT keratinocytes, one of the most sensitive *in vitro* cell models for PLTX [18,28].

## 2. Results

### 2.1. Evaluation of PLTX Binding to HaCaT Cells

**Figure 1:**
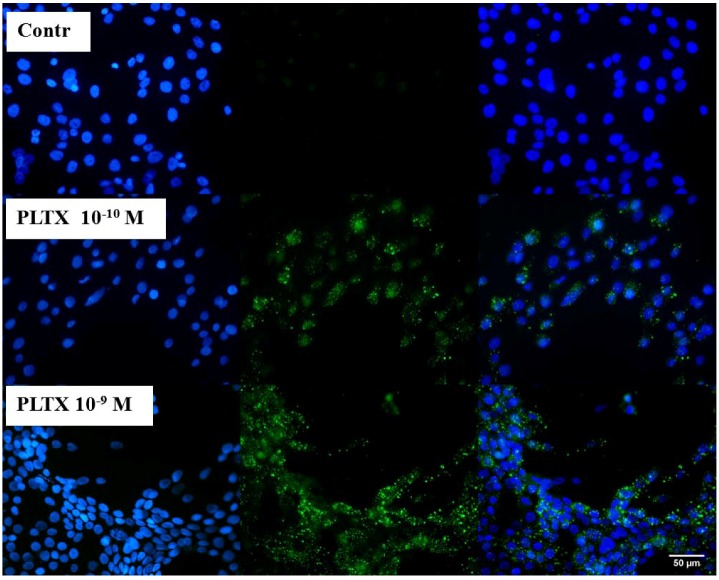
Immunocytochemical analysis of Palytoxin (PLTX) binding on HaCaT cells. Cells were exposed to 1.0 × 10^−10^ and 1.0 × 10^−9^ M PLTX for 10 min, and bound PLTX was detected by mouse monoclonal PLTX antibody and anti-mouse IgG secondary antibody AlexaFluor488-conjugate (green fluorescence). Nuclei were stained with 1 μg/mL DAPI (blue fluorescence). In the third panel, a merge of the green and blue fluorescence is shown. Images were taken with an epifluorescent microscope (Eclipse E800, Nikon) at a 40× magnification.

To verify the feasibility of investigating the binding of PLTX to intact cells, immunocytochemical analyses were performed as a qualitative assay. Cells were exposed for 10 min to 1.0 × 10^−10^ and 1.0 × 10^−9^ M PLTX and washed twice with PBS in order to remove the unbound toxin. PLTX binding was recognized by a mouse monoclonal PLTX antibody and, subsequently, by a secondary anti-mouse antibody AlexaFluor488-conjugate (green fluorescence). Nuclei were then stained with DAPI (blue fluorescence) and images taken by an epifluorescence microscope. In Figure 1, toxin-exposed cells are compared to the untreated control cells. The presence of 1.0 × 10^−10^ and 1.0 × 10^−9^ M PLTX was easily detected by the green signal that, by contrast, was absent in the untreated controls (no PLTX exposure, followed by PLTX antibody and anti-mouse secondary antibody). As internal control, the secondary anti-mouse IgG antibody was used and gave no nonspecific signal (data not shown).

**Figure 2:**
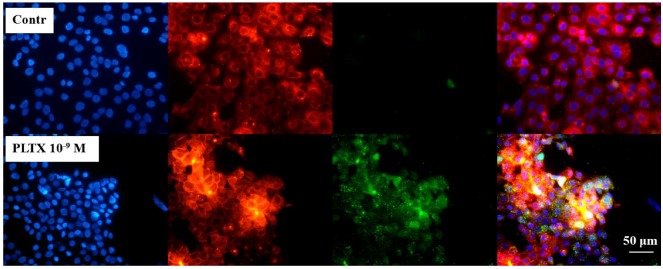
Immune localization analysis for PLTX on HaCaT cells. Cells were exposed to 1.0 × 10^−9^ M PLTX for 10 min and PLTX bound detected by mouse monoclonal PLTX antibody and anti-mouse IgG secondary antibody AlexaFluor488-conjugate (green fluorescence). Nuclei were stained with 1 μg/mL DAPI (blue fluorescence) and cell membrane with 1.0 × 10^−5^ M DiL (red fluorescence). The fourth panel represents a merge of the green, blue and red fluorescence. Images were taken by epifluorescent microscope (EclipseE800, Nikon) at a 40× magnification.

In order to localize the toxin at cellular level, preliminary localization experiments were performed. Cells were exposed for 10 min to 1.0 × 10^−9^ M PLTX and, after two washes with PBS, the toxin detected, as described above (green fluorescence). Nuclei were then stained with 1 μg/mL DAPI (blue fluorescence) and plasma membrane stained with 1.0 × 10^−5^ M DiL (red fluorescence). In Figure 2, the three fluorescences are shown alone and after merging (fourth panel). Cells exposed to the toxin displayed a marked green fluorescence (PLTX) that was almost completely overlapped to the red one (membrane), suggesting the toxin binding on the cell surface.

### 2.2. Characterization of PLTX Binding to HaCaT Cells

**Figure 3:**
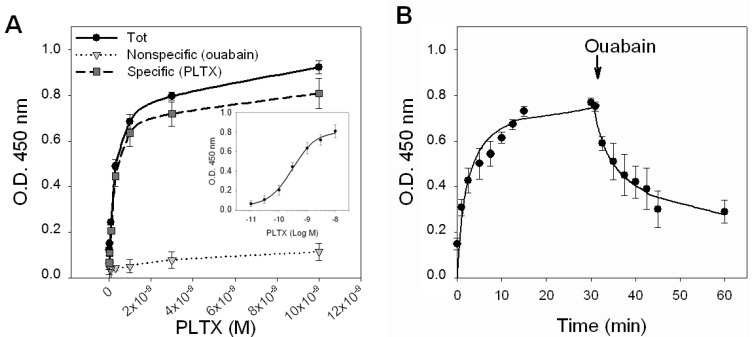
PLTX binding to intact HaCaT cells. (**A**) Saturation experiments: Cells were exposed for 10 min to PLTX (1.0 × 10^−11^–1.0 × 10^−8^ M) and the amount of PLTX bound evaluated by cellular ELISA assay. Total binding of PLTX is represented by black dots (●), nonspecific binding, given by PLTX binding in presence of 1.0 × 10^−3^ M ouabain, is represented by gray triangles (▼) and the specific binding of PLTX, obtained subtracting the nonspecific binding from the total one is represented by gray squares (■). Specific binding is shown in a semi-logarithmic scale in the inner panel. (**B**) Time course of association and dissociation of specific binding of PLTX to intact HaCaT cells. In association experiments, cells were exposed up to 30 min to 3 × 10^−10^ M PLTX at the time intervals indicated. Nonspecific binding was determined in the presence of 1 × 10^−3^ ouabain (OUA). In dissociation experiments, cells were equilibrated with 3 × 10^−10^ M PLTX for 30 min at 37 °C. Ouabain (1 × 10^−3^ M) was rapidly added and specific binding determined at the time intervals indicated. Data are reported as the mean ± SEM of three experiments performed in triplicate.

To characterize the binding of PLTX to HaCaT cells, saturation experiments were performed, exposing intact HaCaT cells to increasing toxin concentrations (1.0 × 10^−11^–1.0 × 10^−8^ M) for 10 min at 37 °C, and a cellular immunoenzymatic (ELISA) assay was carried out using a PLTX monoclonal antibody to detect the toxin binding. Longer incubation times were not attempted, due to the high cytotoxicity of the ligand. Nonspecific binding was measured in the presence of 1.0 × 10^−3^ M OUA, added 10 min before toxin exposure. Figure 3A shows the saturation curves of the total binding, of the nonspecific binding (1.0 × 10^−^^3^ M OUA) and of the specific binding obtained by subtracting the nonspecific binding from the total binding. The curve of the specific binding is shown in a semi-logarithmic scale in the inner panel of Figure 3A. From the analysis of the specific binding curve performed by nonlinear regression, a single binding site was found with a *K_d_* value equal to 2.6 ± 0.3 × 10^−10^ M.

The kinetics of PLTX binding was then investigated by exposing the cells to a PLTX concentration near to the *K_d_* value (3.0 × 10^−10^ M) for increasing time intervals (Figure 3B). The association curve was obtained exposing the cells to PLTX up to 30 min. Under this condition, PLTX was found to quickly bind to HaCaT cells, reaching equilibrium within 10–15 min. An association rate constant (*K*_on_) of 1.297 ± 1.068 × 10^8^ [M^−1^ s^−1^] was estimated. After 30 min of continuous exposure to 3.0 × 10^−10^ M PLTX, dissociation experiments were carried out adding the displacer OUA (1.0 × 10^−3^ M) at increasing time intervals, up to 30 min. The dissociation of 50% of the tracer occurred within 12 min, but was incomplete after 30 min (36% of PLTX binding was still detected). The estimated dissociation rate constant (*K*_off_) was of 0.119 ± 0.015 [s^−1^]. An independent estimation of the dissociation constant of 9.2 ± 8.5 × 10^−10^ M was obtained from the relationship *K_d_* = *K*_off_/*K*_on_.

### 2.3. Competition Experiments: Interaction between PLTX and OUA for the Binding Site on HaCaT Cells

**Figure 4:**
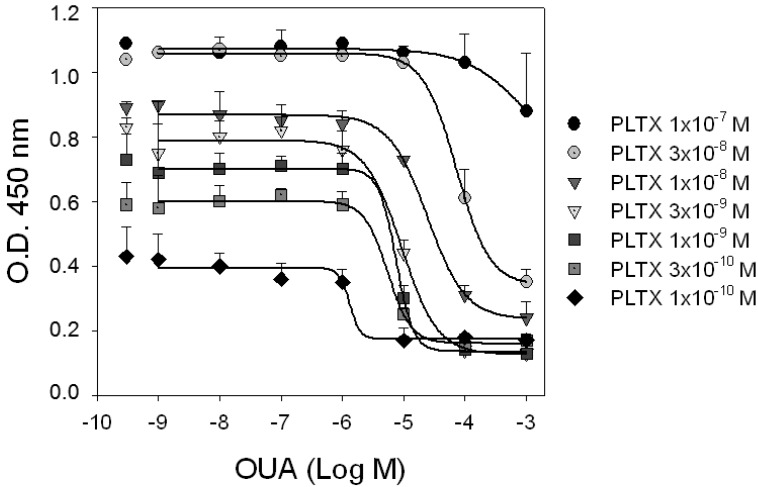
Competition experiments. HaCaT cells were exposed for 10 min to increasing concentrations of OUA in presence of different concentrations of PLTX. Curves represent displacement of PLTX (1.0 × 10^−10^–1.0 × 10^−7^ M) by OUA. Single dots represent binding of PLTX in absence of OUA. Data are reported as the mean ± SEM of three experiments performed in triplicate.

To investigate the mechanism of interaction between PLTX and OUA at the PLTX binding site, competition experiments were carried out exposing HaCaT cells to increasing concentrations of OUA (1.0 × 10^−9^–1.0 × 10^−3^ M) in presence of the tracer ligand PLTX (1.0 × 10^−10^–1.0 × 10^−7^ M). As shown in Figure 4, OUA displacement of PLTX binding was detectable at OUA concentrations starting from 1.0 × 10^−6^ M. The ability of OUA to inhibit PLTX binding did not significantly change in the tracer concentrations ranging from 0.1 to 3.0 × 10^−9^ M PLTX (*p* > 0.05). Indeed, IC_50_ values of OUA (concentrations inhibiting PLTX binding by 50%) were similar and equal to 1.3 ± 12.8, 5.5 ± 1.6, 7.5 ± 3.0 and 9.3 ± 1.0 × 10^−6^ M against 0.1, 0.3, 1.0 and 3.0 × 10^−9^ M PLTX, respectively. On the contrary, OUA displacement potency against 1.0 and 3.0 × 10^−8^ M PLTX decreased with IC_50_ values of 3.4 ± 0.4 and 7.3 ± 0.3 × 10^−5^ M, respectively, and were found to be significantly different between them (*p* < 0.001) and among the IC_50_s of the lower PLTX concentrations (*p* < 0.001). Moreover, the displacement binding curves did not appear to be complete, a hallmark of allosteric interaction. 

### 2.4. Inhibition of PLTX Cytotoxicity by OUA

**Figure 5:**
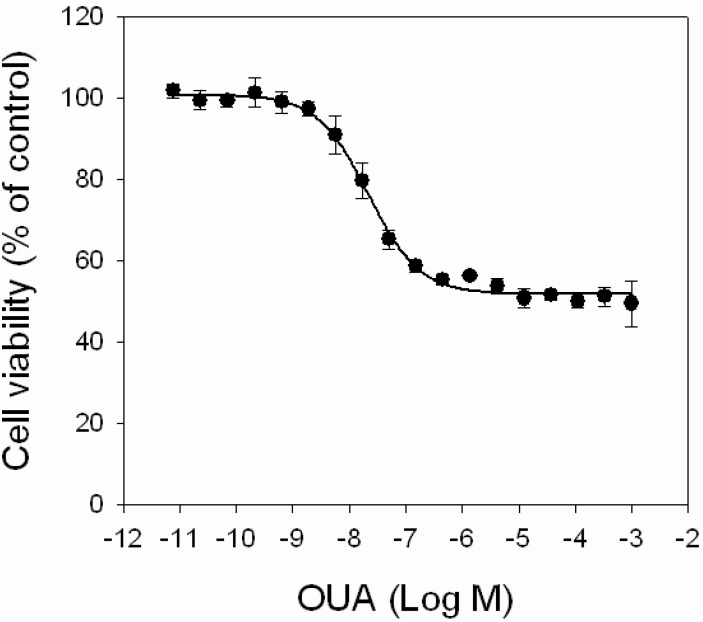
Cytotoxic effects of OUA. HaCaT cells were exposed to increasing concentrations of OUA (1.0 × 10^−11^–1.0 × 10^−3^ M) for 4 h before performing MTT assay. Data are reported as % of control and represent the mean ± SEM of four independent experiments performed in triplicate.

First, the effect of OUA itself on cell viability was evaluated by MTT assay, exposing HaCaT cells for 4 h to increasing concentrations of OUA (1.0 × 10^−11^–1.0 × 10^−3^ M). OUA induced a concentration-dependent cytotoxic effect in the 1.0 × 10^−9^–1.0 × 10^−6^ M range (EC_50_ value of 2.0 ± 0.2 × 10^−8^ M; Figure 5) that reached a plateau at 1.0 × 10^−5^ M, with a maximal reduction of cell viability of 45%. Higher OUA concentrations did not further reduce cell viability.

**Figure 6:**
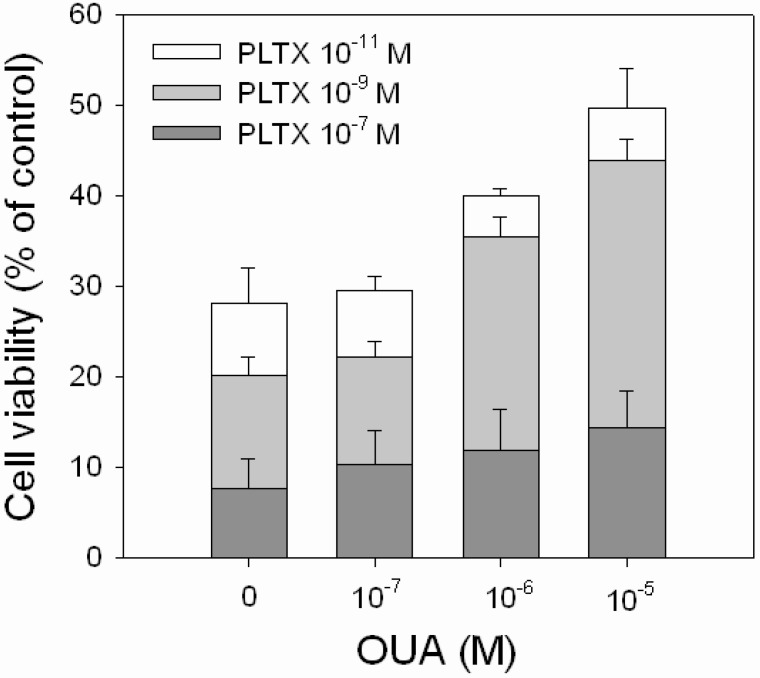
Inhibitory effect of OUA on PLTX cytotoxicity. HaCaT cells were exposed to OUA (1.0 × 10^−7^–1.0 × 10^−5^ M) for 30 min and then to PLTX (1.0 × 10^−11^–1.0 × 10^−7^ M) for 4 h before performing the MTT assay. Data are reported as % of control and represent the mean ± SEM of four independent experiments performed in triplicate.

OUA ability to reverse the cytotoxic effect of PLTX was evaluated exposing the cells to PLTX alone (1.0 × 10^−11^, 1.0 × 10^−9^ and 1.0 × 10^−7^ M) or to PLTX in the presence of OUA concentrations that significantly affected cell viability (1.0 × 10^−7^, 1.0 × 10^−6^ and 1.0 × 10^−5^ M). A partial, but significant, reduction of PLTX-induced cytotoxicity was observed in the presence of 1.0 × 10^−6^ and 1.0 × 10^−5^ M OUA (Figure 6). In the presence of 1.0 × 10^−6^ M OUA, the increase of cell viability was equal to 18% and 15% with respect to 1.0 × 10^−11^ M (*p* < 0.001) and 1.0 × 10^−9^ M (*p* < 0.001) PLTX. In the presence of 1.0 × 10^−5^ M OUA, the increased cell viability was equal to 25% and 23% with respect to 1.0 × 10^−11^ M (*p* < 0.001) and 1.0 × 10^−9^ M (*p* < 0.001) PLTX, respectively. No inhibitory effects were observed in the presence of 1.0 × 10^−7^ M OUA, as well as at OUA concentrations that *per se* did not affect cell viability (1.0 × 10^−11^ and 1.0 × 10^−9^ M, data not shown).

### 2.5. Discussion

The high cytotoxicity of PLTX has been reported in several studies [18,19,29,30,31]. In general, PLTX reduces cell viability with high potency, often after short periods of exposure. It is widely accepted that the prominent mechanism of action of PLTX is based on interaction with the Na^+^/K^+^-ATPase, which is consequently transformed into a nonspecific cationic channel. Thus, it is reasonable to infer that the strong cytotoxicity could be dependent on a high affinity interaction with the pump. This study was carried out to characterize PLTX binding to cultured intact cells and to investigate the ability of OUA to compete for this binding and, thus, to reduce PLTX cytotoxicity. To this end, we choose the HaCaT cell line, which is one of the cell models most sensitive to the cytotoxic effects of PLTX [18,28].

PLTX binding to intact HaCaT cells was first demonstrated by immunocytochemical analysis after 10 min exposure. Preliminary immune localization experiments suggested that the binding site for the toxin is located within the plasma membrane. However, confocal microscopy experiments are needed to confirm the cellular localization of PLTX binding sites. These results are consistent with the notion that PLTX exerts its effects on cells by binding to a cell membrane-located target, presumably the Na^+^/K^+^-ATPase. 

Taking advantage of the ability of PLTX monoclonal antibodies to detect and label PLTX bound to intact cultured HaCaT cells, binding experiments were carried out using a PLTX monoclonal antibody-based cellular ELISA. Saturation experiments were performed using OUA to define nonspecific binding. The use of OUA was supported by its inhibitory effect on PLTX-induced cytotoxicity, as reported by several groups [15,16,17,18,19]. Moreover, the ability of OUA to reduce the PLTX effects has been already exploited to characterize PLTX binding to Na^+^/K^+^-ATPase. Indeed, it has been reported that OUA inhibits PLTX binding to human erythrocytes with an IC_50_ of 3 × 10^−9^ M [26]. Conversely, [3H] ouabain was found to be displaced by PLTX with a *K_i_* of 3 × 10^−11^ M from human erythrocytes [26] and with an IC_50_ value of 2.9 × 10^−8^ M from purified porcine Na^+^/K^+^-ATPase [27]. Furthermore, OUA has been recently used to develop the protocol of a new receptor (*i.e.*, Na^+^/K^+^-ATPase)-based detection method for PLTX, based upon fluorescence polarization [32].

Using a monoclonal antibody-based cellular ELISA assay, we found that PLTX binding to intact HaCaT cells was saturable and reversible, with a single high affinity-binding site and a calculated binding constant (*K_d_*) value of 2.6 × 10^−10^ M. However, when kinetic experiments were carried out, after 30 min of exposure to OUA, the dissociation of PLTX binding was not complete. This result could suggest the presence of an additional, OUA-insensitive, PLTX binding site or the possibility that PLTX and OUA do not share the same binding site on Na^+^/K^+^-ATPase, as previously suggested [17,21,22].

Interestingly, in a recent study, Sandtner and co-workers described two mutually exclusive binding sites for OUA on the Na^+^/K^+^-ATPAse: a high affinity binding site at the inner end of the permeation pathway and an extended low affinity binding site [33]. Since PLTX molecular weight is 10-times higher than that of OUA, a pre-incubation with the toxin, as performed in the kinetic experiments, could hinder OUA binding to its internal high affinity site. Under this condition, OUA cannot be considered an appropriate choice to evaluate PLTX-related nonspecific binding. However, PLTX itself cannot be used to define nonspecific binding in an ELISA-based binding assay.

On the basis of its well-known ability to bind to the Na^+^/K^+^-ATPase, OUA has been extensively used to inhibit some of the biological activities of PLTX, such as hemolysis of red blood cells and cytotoxicity. This indirectly supports the involvement of the pump in the molecular mechanism of the toxin. However, whereas PLTX is believed to stabilize the pump in an open conformation dependent on the phosphorylation state [34], OUA seems to block it in a closed conformation state [35]. These two different mechanisms of interaction with the same target could be reasonably ascribed to the binding to different sites on the same target. However, alternative explanations are possible, such as different affinities, different efficacy (agonism *vs.* antagonism or inverse agonism) or interaction with allosteric modulatory sites. Hence, the ability of OUA to antagonize PLTX binding and, as a consequence, PLTX effects could result from negative allostery. Allosteric modulation can produce either surmountable or insurmountable effects and can reach a maximum when the allosteric site is saturated, depending on the ligand and/or the cellular environment. An allosteric mechanism could thus explain the inability of OUA to completely reverse PLTX toxic effects observed in HaCaT [18] and in Caco-2 cell lines [19].

The possibility that OUA can act as an allosteric modulator of PLTX binding was thus investigated performing competition experiments using different concentrations of PLTX as a tracer and scalar concentrations of OUA (1.0 × 10^−9^–1.0 × 10^−3^ M) as displacer. The results suggested a more complex scenario. Indeed, OUA appeared to act as negative allosteric modulator of PLTX binding, since the displacement curves were shifted to the right and, at high PLTX concentrations, did not reached the displacement levels obtained for lower PLTX concentrations. However, notwithstanding their tendency to increase, OUA IC_50_ values against low PLTX concentrations (0.1–3 × 10^−9^ M) were found to be not statistically different, a result that could suggest a noncompetitive antagonism. On the basis of these results and considering that OUA exhibits a low, micromolar potency in displacing PLTX concentrations as low as 1.0 × 10^−10^ M, despite its nanomolar affinity towards the Na^+^/K^+^ ATPase [27], it seems reasonable to speculate that OUA and PLTX do not share the same binding site. Furthermore, very high OUA concentrations (10^−3^ M) were needed to ensure PLTX displacement. These results could derive from differences in binding kinetics (association/dissociation rates) between OUA and PLTX, from the presence of OUA-insensitive binding sites or both. However, further experiments are required to confirm this conclusion.

We finally attempted to find a relationship between OUA as a PLTX binding antagonist/modulator and OUA as antagonist of PLTX-induced cytotoxic effects. OUA *per se* was found to partially reduce HaCaT cells viability. The cytotoxic effect of OUA is believed to be not directly related to its classical action as a Na^+^/K^+^-ATPase inhibitor, but to be rather associated with the induction of the Ras-ROS signal transduction pathway [36]. Notwithstanding its intrinsic cytotoxicity, OUA was able to significantly reduce PLTX-induced decrease of cell viability. However, OUA only partially prevented the PLTX cytotoxic effect and was ineffective against high PLTX concentrations (10^−7^ M). Moreover, an incomplete consistency between the ability of OUA to affect PLTX-induced cytotoxicity and to displace PLTX binding was found. Inhibition of PLTX cytotoxicity was observed in the presence of 10^−5^ M OUA, a concentration that also affected PLTX binding, supporting an antagonism at the binding site level. On the contrary, 1.0 × 10^−6^ M OUA significantly reversed PLTX toxic effect, but did not displace PLTX binding, strengthening the hypothesis of the existence of both OUA-sensitive and OUA-insensitive PLTX-induced mediated cytotoxic activities, possibly mediated by different or only partially overlapping binding sites. However, further experiments are required to confirm this conclusion, possibly carried out on OUA-insensitive cell models, such as the BE(2)-M17 neuroblastoma cell line [31].

In conclusion, this study demonstrates the presence, on intact HaCaT cells, of a high affinity binding site for PLTX located on the cell surface that appears to be partially insensitive to OUA and partially modulated by OUA in a complex manner (noncompetitive antagonism/negative allosterism). This hypothesis could explain the inability of OUA to totally prevent PLTX-induced cytotoxic effects.

## 3. Experimental Section

### 3.1. Chemicals

Palytoxin, isolated from *P. tuberculosa*, was purchased from Wako Pure Chemical Industries Ltd. (Osaka, Japan; lot number WKL7151, purity >90%). HaCaT cell line was purchased from Cell Line Service (DKFZ, Eppelheim, Germany), and all cell culture reagents were from Euroclone (Milan, Italy).

All the other reagents of analytical grade were purchased from Sigma-Aldrich (Milan, Italy) if not otherwise specified.

### 3.2. HaCaT Cells Culture

HaCaT cells were cultured in DMEM supplemented with 10% fetal bovine serum (FBS), 1.0 × 10^−2^ M l-Glutamine, 1.0 × 10^−4^ g/mL penicillin and 1.0 × 10^−4^ g/mL streptomycin at 37 °C in a humidified 95% air/5% CO_2_ atmosphere. Cell passage was performed 2 days post-confluence, once per week. 

All the experiments were performed between passage 48 and 73.

### 3.3. Immunocytochemical Analysis

Cells (2 × 10^5^ cells/well) were seeded in 24-well plates. After 2 days in culture, cells were exposed to 1.0 × 10^−10^ and 1.0 × 10^−9^ M PLTX for 10 min. Cells were then washed with PBS, fixed for 30 min in 4% *p*-formaldehyde (PFA) and blocked for 30 min in TBB buffer (Tris-HCl 50 mM, NaCl 0.15 M, 2% BSA and 0.2% Tween 20, pH 7.5) containing 10% horse serum. PLTX binding was then detected by 1:500 (final concentration 2 μg/mL) murine monoclonal antibody 73D3 against PLTX [37] and by 1:200 (final concentration 10 μg/mL) secondary anti-mouse IgG antibody conjugated with AlexaFluor488 (Invitrogen, Milano, Italy) in TBB buffer. Nuclei were detected by 1 μg/mL DAPI for 5 min in PBS. Images were taken by an epifluorescent microscope (Eclipse E800, Nikon, Kingston, England, UK).

For immunocolocalization analysis, after DAPI staining, cells were incubated with 10^−5^ M DiL for 10 min, and images were taken by an epifluorescent microscope (Eclipse E800, Nikon, Milano, Italy) at a 40× magnification; scale bars were calculated using ImageJ software (version 1.45s).

### 3.4. Binding Experiments

Cells (1 × 10^4^/well) were seeded in 96-well plates and maintained in culture for 3 days. After 10 min exposure to PLTX and/or OUA, cells were washed twice with PBS and fixed for 30 min with 4% PFA. Cells were then blocked for 30 min in TBB buffer containing 10% horse serum and the toxin detected by 2 μg/mL mouse PLTX mAb for 1 h at 37 °C. Cells were then washed three times with PBS containing 0.1% Tween 20 (PBS/Tw) followed by three washes with PBS. PLTX mAb was detected by exposing the cells to 1:3000 HRP conjugated secondary antibody against mouse IgG (DakoCytomation; Milan, Italy) for 1 h at 37 °C. After three washes with PBS/Tw and three washes with PBS, the colorimetric reaction was started by adding 60 μL 3,3′,5,5′-Tetramethylbenzidine (TMB) substrate for 15–20 min and stopped by adding 30 μL H_2_SO_4_ 1 M. The absorbance was read at 450 nm by a Spectra^®^ photometer (Tecan Italia; Milan, Italy).

The time course of association of 3 × 10^−10^ M PLTX specific binding to HaCaT cells was determined by exposing the cells at increasing time intervals to PLTX up to 30 min at 37 °C. In dissociation studies, cells were equilibrated for 30 min with 3 × 10^−10^ M PLTX. Ouabain (10^−3^ M) was than rapidly added to the incubation mixture in a 1:10 ratio (time 0) and the specific binding determined at increasing time intervals up to 30 min. 

### 3.5. MTT Assay

Cells were seeded in 96-well plates at a density of 3 × 10^3^ cells/well and after 72 h in culture exposed to PLTX and/or OUA. Cells were then washed and wells refilled with fresh culture medium containing 0.5 mg/mL 3-(4,5-Dimethylthiazol-2-yl)-2,5-diphenyltetrazolium bromide (MTT). After 4 h, the insoluble crystals were solubilized by 200 μL/well DMSO, and the absorbance was measured by an Automated Microplate Reader EL 311s (Bio-Tek Instruments, Winooski, VT, USA) at 540/630 nm. Data are reported as % of control and are the mean ± SEM of 4 independent experiments performed in triplicate.

### 3.6. Statistical Analysis

Binding constants (*K_d_*, *K*_on_ and *K*_off_) were calculated using the GraphPad software, version 6.0 (Prism GraphPad, Inc.; San Diego, CA, USA). *K_d_* constant was calculated by a one-site binding hyperbola nonlinear regression analysis. *K*_obs_ and *K*_off_ were calculated from the results of the association/dissociation experiments, respectively, by fitting the data to a one-phase exponential association/decay nonlinear regression analysis. *K*_on_ was calculated from the equation *K*_on_ = (*K*_obs_ − *K*_off_)/[PLTX].

IC_50_ values were calculated by 4-parameters nonlinear regression analysis using the SigmaPlot software (Jandel Scientific; Erkrath, Germany).

Data are the mean ± SEM of at least three different experiments performed in triplicate. Data were analyzed by two-way ANOVA, followed by Bonferroni’s post-test (Prism GraphPad, Inc.; San Diego, CA, USA), and significant differences were considered at *p* < 0.05. IC_50_ values were analyzed by one-way ANOVA, followed by Bonferroni’s post-test (Prism GraphPad, Inc.; San Diego, CA, USA), and significant differences were considered at *p* < 0.05.

## 4. Conclusions

In conclusion, PLTX binding to intact HaCaT cells was characterized by an anti-PLTX monoclonal antibody-based cellular ELISA. The binding was saturable and reversible, with a *K_d_* of 2.6 ± 0.3 × 10^−10^ M. Kinetic experiments revealed that PLTX binding dissociation was incomplete, suggesting an additional, OUA-insensitive, PLTX binding site. Moreover, competitive experiments suggested that OUA acts in a complex manner: as a non-competitive antagonist against low PLTX concentrations and as a negative allosteric modulator against high PLTX concentrations. These results could explain the inability of OUA to totally prevent PLTX-induced cytotoxic effects.
